# Hospital Meetings

**Published:** 1901-07-13

**Authors:** 


### The Institutional Workshop.

### HOSPITAL MEETINGS.

### NATIONAL HOSPITAL FOR THE PARALYSED AND EPILEPTIC.

The Committee of Inquiry's Recommendations: Important Administrative Changes Suggested.

A special general meeting of Governors and subscribers of the National Hospital for the Paralysed and Epileptic (Albany Memorial) was held on Saturday, the 6th inst., in the Hospital, Queen's Square, Bloomsbury, W.C., to receive and consider the report of the Committee of Inquiry appointed pursuant to the resolutions passed at the special general meeting held on March 23rd last. Mr. John Pearman was voted to the chair, and the other members of the Board of Management present were Sir Gerald FitzGerald, Mr. Colin F. Campbell, Mr. George W. E. Russell, and Mr. A. L. Wheeler. Mr. Burford Rawlings, the secretary-director, also occupied a seat at the board table.

The Committee of Inquiry consisted of Sir Edward Fry (Chairman), Lord Wolverton, Sir W. W. Karslake, Mr. Timothy Holmes, and Mr. Cecil H. Russell, with Mr. J. Danvers Power as hon. secretary. These gentlemen having received the thanks of the meeting for their valuable services in conducting the investigation,

Mr. Melvill Green proceeded to move the adoption of the Committee's report. He said the Committee, of which he was a member, was responsible for the proposition, but he alone would be responsible for what he might say in support of it. In the first place he intended to read certain portions of the report so as to justify the course which he would ask the Governors to take. In Clause 13 the Committee said:—"We have inquired into the truth of the several allegations contained in the statement of the medical staff, and we find that some of the most important of them are true, that some have a foundation of fact but are exaggerated, and that others are unsupported by any sufficient evidence." Then in Clause 53 they said:—"We can find no foundation for the fears entertained that the character of the hospital as a religious and philanthropic institution will be imperilled by the presence of two medical men on the Board of Management, and we are therefore quite unable to concur with the present board when they characterise the demands of the medical staff as 'encroachments calculated to destroy the religious and philanthropic character of the hospitals, and to subordinate to purely professional objects institutions maintained by the benevolent for benevolent purposes.'" In Clauses 50 and 51 the Committee said: "Another of the objections urged to the direct representation of the medical staff on the Board of Management is that it will give undue influence to the scientific element in the hospital, and will tend to subordinate the immediate benefit of the patients to the pursuit of medical knowledge; and in support of this view it was suggested to us that there was ground for a suspicion that in one case the sufferings of the patient had been prolonged in the interest of medical science. We investigated the only case which could be adduced as in any way supporting this suggestion, and we find that the child in question was in fact retained in the hospital by the Board of Management in accordance with a resolution of the medical committee that she ought to be retained 'in her own interest.' Nothing was adduced to impugn the opinion of the medical committee or the action of the Board of Management, and we dismiss the suspicion suggested as unfounded." (Applause.) Then the Committee said in Clause 14: "It is our opinion that the defects in management, to which attention was drawn by the statement, were deserving of notice and correction; but that they were never sufficient in magnitude and frequency to render the treatment in the Hospital otherwise than good as a whole; and further that the Medical Committee were, having regard to the position of things in the early part of last year, justified in addressing their statement to the Governors and Members of the Hospital without previous communication with the Board of Management." Those four passages seemed to him to show what he ventured to say last August, namely, that both sides had exaggerated the position of affairs, but that there was truth in their contentions. But they were very satisfactory statements to them as Governors, because they showed that fears were unfounded and that the amount of mischief done to the hospital was not of an important character—it was trivial. There was one other satisfactory topic to which he wished to refer, and that was finance. Whenever it was mentioned that there were irregularities in connection with the finances of any public institution people were apt to get frightened. Well, there were irregularities in this case. There ought to be three trustees of the funds, and there were only two. A sum of money had also been placed to one account that ought to have been placed to another; that was to say, it had been invested in what it ought not to have been invested. But the matter of finance was in the popular sense absolutely right. (Applause.) There was nothing wrong with the exception of merely technical matters which it was quite proper to call irregularities. With that exceedingly satisfactory preface he must come to the business of the day. He ventured to think that there hardly could be a more momentous occasion in the history of the hospital, because the adoption of the report would undoubtedly effect a very great and important change in its management. (Applause.) He was told that there would be no division upon the matter, and therefore he trusted the report would be adopted unanimously. (Hear, hear.) He thought such a decision was inevitable. In Clause 66 the Committee said: "We are of opinion that the supervision of the affairs of the hospital by the Board of Management has not been adequate to the needs of the institution." He thought that was so, and by adopting the report they would say so. (Applause.) The rules provided that there should not be less than twelve members of the board. There were twelve, but one or two members, in consequence of their personal distinction or past services—in fact in consequence of excellent reasons—had been retained upon the board when they were not able to give anything like proper attention to its affairs. The effect had been to reduce the working board to less than twelve, which was contrary to the rules. This was one of the many things that would have to be altered. (Applause.) The words which he had just quoted really related to the members of the board who had been most frequent in their attendance. He thought he would be quite within the mark if he said that out of the twelve members of the board not more than eight had attended with so much regularity as one would have expected of them. This brought him to the two greatest recommendations of the report. In Clause 55 the Committee recommended "that the Board of Management be increased by the addition of two members chosen by the Medical Committee from the medical staff." (Applause.) Then in Clause 48 they said: "We are of opinion that the mode of representation desired by the medical staff is a very proper one, and is the smallest concession which is reasonable." (Renewed applause.) By adopting the report they would determine that that should be the rule of the hospital henceforth. The rules would have to be altered at a subsequent meeting, and therefore the second motion which was to be submitted was that a committee be appointed to prepare for that. By adopting the report he hoped it was understood by everybody that it put an end to all controversy on the question whether the staff should be represented on the Board of Management by two of its members. (Applause.) Then in Clause 79 the Committee expressed the opinion that the office of General Director in the hospital should be abolished. They took it for granted that there was such an office, although he was not at all sure about it himself. There was nothing in the rules to create it. At any rate he did not think that on the resignation of Mr. Burford Rawlings, which they had already in their hands, there was any office left. However, the Committee recommended that the office should be abolished, and that the hospital should be carried on in one or other of two alternative ways, but they did not say which. That showed the necessity of appointing a committee to report to the Governors which alternative or what course should be adopted to bring about the abolition of the office of General Director. But if they adopted the report they would again finally and conclusively determine that there should be no General Director of the hospital. (Applause.) Those were the two great things that had been the subject of controversy for he did not know how many years—fifteen at least—and the cause of all the friction and unpleasantness. The principal object of the report was to get rid of those two great subjects of controversy. In Clause 67 the Committee stated that "the action alike of the Secretary-Director and of the Board of Management has tended to hinder the free access of communications from the medical officers to the board." That was a finding of fact. In illustration of the attitude on this point assumed by the secretary-director, the committee referred to an incident which occurred in 1890. "A member of the medical staff was required by Mr. Rawlings to give certain explanations as to his absence from the hospital, and he, desiring to communicate directly with the board, enclosed to Mr. Rawlings a sealed letter for the chairman at the next board meeting. Mr. Rawlings returned the letter, and wrote that, 'apart from all personal feelings, I cannot think that I am justified in acquiescing in an effacement of the position the Board of Management have done me the honour to assign me.'" In the next paragraph a very remarkable incident was recorded, but before reading it he wished to say that nothing in the report struck him perhaps more forcibly than the forbearance of the staff. (Applause.) They made a complaint to the Board of Management, and the board found it well founded, and then at a subsequent meeting they sent for the staff, not to get their opinions or to consult them, but to reprimand and scold them. The minute of the board was as follows:—"The chairman referred to the matter brought under the notice of the board at their last meeting and said that the board had thought it desirable to invite the members of the staff to meet them in order that the board might have an opportunity of saying that as the secretary-director was entrusted with the control and general direction of the hospital and with the authority of the Board of Management in their absence, it was desirable he should be informed at once if anything came to the notice of the members of the staff or others which appeared detrimental to the interests of the hospital or to call for action. The board were further of opinion that when a need arose for inquiry and investigation it should be made by the secretary-director and not by any other officer whether medical or lay." The minute did not say what happened, but apparently the staff went away with their tails between their legs. (Laughter.) With these facts before them it was impossible to have any doubt about the conclusion. Then in Clause 77 the committee said:—"It should be added in justice to Mr. Rawlings that the course he pursued in the case of this child, and in most of, or perhaps all, his dealings with the medical staff, received the approval and support of the Board of Management; that, in our opinion, he was throughout acting in the manner which he thought to be his duty, and which was in the spirit of the instructions which he received from the board; and that on them rather than on him rests the responsibility for the attitude he assumed towards the medical staff." (Applause.) His (the speaker's) own opinion coincided entirely with that expressed by the committee. There was nothing to blame Mr. Rawlings for excepting that he did not perceive the falseness of his position. He acted according to his directions from the board, and the blame—if blame there be—rested upon the board, and chiefly upon those who had been most frequent in their attendance. (Applause.) It was evident that after the abolition of the office of general director Mr. Rawlings would no longer be able to fill that position, and having been secretary and director for so many years it was hardly to be expected that he would retain the office of secretary. Therefore he (the speaker) was not surprised at receiving a letter from him announcing his intention to resign that office. Personally he felt very sorry for Mr. Rawlings. He had devoted the best part of his life to the service of the hospital and done a great deal of excellent work for it. (Applause.) Then it came to pass that they were obliged to take such a course that he must retire. ("No.") Well, he thought it was essential, and although he felt very sorry about it he must ask the meeting to adopt the report unflinchingly. In the presence of eminent surgeons he did not like to use an illustration, but it was sometimes necessary to cut off the right arm to save the body, and he thought if that were necessary here they would have to do it. In his opinion it was necessary. The source of all the mischief in connection with this hospital for many years past was, that it had been talked of popularly as a "one-man hospital." One could not be blind to the fact that there was a good deal of censure of the board in what he had quoted. He understood the members who were present would give an explanation, and perhaps it would be found satisfactory, although the one they had already given did not appear to have been satisfactory to the Committee of Inquiry. He did not wish to suggest that the members of the board should resign. The Governors had no power to do so. It was true they could elect two members if three months before the annual meeting—which they did not know of—they gave notice of their intention to propose two persons. Thus in the course of six years they could change the board, and in the course of four years they could change the majority. (Laughter.) That was one of the rules that would have to be dealt with; in fact, the rules would have to be revised from beginning to end. There were several other matters which must be dealt with by the Governors some day, such as the payment of out-patients, voting by proxy, the insufficiency of the nursing staff, etc. Many of these things would cure themselves by the presence upon the board of two members of the medical staff. (Applause.)

Sir JAMES CRICHTON-BROWNE seconded the motion, and in doing so said it seemed to him that the Committee had performed the duty assigned to it in a most thorough and satisfactory manner. (Applause.) Those who had carefully read the report could not fail to arrive at the conclusion that the inquiry instituted had been searching, that the judgment exercised had been impartial, and that the recommendations made were sound and practical. It was impossible to thoroughly grasp the significance of the report without comparing it paragraph by paragraph with the statement that the medical staff made in May, 1900, and with the report of the sub-committee of managers appointed to inquire into the complaints made in that report. Any such minute and detailed comparison, however, beyond that which Mr. Melvill Green had presented, was not only unnecessary, but undesirable, he thought, for it could not now be denied that the main contentions of the medical staff had been amply justified—(hear, hear)—and that they had correctly indicated the sources from which all difficulties in the administration of the hospital had arisen. He said he did not think such a comparison necessary or desirable, because he believed that they had now arrived at the hour of pacification, and as far as possible they should avoid indulging in recrimination. (Hear, hear.) There should be no stirring up of the sediments of controversy, and all who were interested in the hospital should cordially combine to secure for it a peaceful and prosperous career. (Applause.) Mr. Rawlings, who, he supposed, was still acting as the mouthpiece of the Board of Management, had, in proper and dignified terms, acknowledged that there was no appeal from this report, and as there was no appeal there was no room for argument, debate or discussion, and all they had to do was to ensure that the recommendations of the Committee should be fully and faithfully carried out, and that there should be no slackness or backsliding; and in order to do that they must secure the co-operation of the Governors, the managers, the medical staff, and all the officials of the hospital. In order that the hospital might be restored to strength and vigour they had to give it support at this critical moment, and that support would be best given by appointing a committee who would thoroughly, efficiently, and effectually carry out the recommendations that had been made. (Applause.) He only wished to add one word, and that was that when the controversy was at its height, he ventured to say on behalf of the medical staff that they cordially recognised the valuable services of Mr. Burford Rawlings to the hospital in his capacity as secretary. (Hear hear.) That day he was sure every member of the medical staff rejoiced to find that in this, as in so many other things, the Committee of Inquiry had adopted their view, and had expressed the hope that in any changes that might ensue upon their report the claims of Mr. Burford Rawlings upon the gratitude of the hospital would not be ignored. (Applause.) He had been, as Mr. Melvill Green had said, a devoted servant of the hospital for 33 years; he had given all his best energies to its service, and he had undoubtedly drawn towards it a large measure of pecuniary support. On his own part as a Governor he would say that during frequent communications Mr. Burford Rawlings, even through all the difficulties that had arisen, had always treated him with the utmost consideration, kindness, and courtesy. But if they owed a great debt of gratitude to Mr. Burford Rawlings, they owed a tenfold debt of gratitude to the medical staff, who had given not only their professional and scientific services to the hospital, but had actually saved it from ruin. Without the action of the medical staff there would have been no Committee of Inquiry; without the Committee of Inquiry there would have been no medical staff; without that committee all those abuses that had been exposed would have gone on and increased, the staff must have inevitably resigned, and the hospital must have sunk to a position that was pitiable to contemplate. (Applause.)

Mr. H. H. Nelson said this was the most important meeting that had ever been held in the history of that noble charity, of which they all had the honour to be supporters, and yet it had been convened on a day and at an hour when it was almost impossible for a great number of the subscribers to attend. (Hear, hear.) The Committee of Inquiry had no doubt taken the greatest pains to carry out the mandate that was given to them, but he did not think the majority of the subscribers would consider it their bounden duty, as they were told, to adopt the report in its entirety without discussion. He for one would be very sorry if the adoption of the report should involve the severing of Mr. Rawlings's connection with the hospital. Moreover, he doubted the wisdom of appointing a medical man to discharge his duties. They were bound to adopt the report, but they were not bound to give a mandate to the board to carry out every recommendation in that report. ("No.")

The report of the Committee was then unanimously adopted.

Mr. Melvill Green next moved: "That a committee be appointed to represent the Governors in making such arrangements as may be necessary for dealing with all matters consequential upon or arising out of the adoption of the previous resolution." That was a rather widely-worded resolution, and, of course, it meant that the Committee would consider the whole management and constitution of the hospital, and bring forward at another meeting of the Governors proposals for altering the rules or such other matters as might require their decision. But he thought the motion went a little further. One of the most important things to be dealt with would be the voting of a pension to Mr. Rawlings. (Hear, hear.) It was quite impossible, of course, for the Governors to meet again before October at the earliest, and they might have to deal with some things which occurred in the interval; at least, that was the construction he put on the resolution, and he thought the board agreed with him that a wide resolution of that character was better.

Sir Sydney Waterlow seconded the resolution, subject to the addition of the words "reporting to the Governors their recommendations and actions." He thought that in altering the constitution of the hospital it would be necessary to have the decision of a meeting of the Governors, and therefore the Committee should certainly report their recommendations and actions to a subsequent meeting.

Mr. Melvill Green accepted the addendum to the resolution.

Sir Sydney Waterlow congratulated the board and the Governors generally upon the position arrived at, after a long and weary, and, to many, a very tiresome and unpleasant discussion, and expressed the hope that the committee to be appointed would frame such a new constitution as would be of permanent benefit to the institution and bring into harmony the staff with the governing body. (Applause.)

Mr. Victor Horsley said he wished to ask a question with regard to the expenses of the enquiry.

The Chairman stated that it was hardly in order to raise that question on the motion now before the meeting.

Mr. Victor Horsley pointed out that the medical staff had not put the hospital to a single halfpenny of expense, whereas the board had involved it, and were involving it, in an expenditure of hundreds of pounds. He therefore wished to know whether the charity was to be taxed or not for the payment of the expenses.

The Chairman stated that the committee to be appointed would deal with that as with other matters in the report. The Committee of Inquiry had made certain recommendations with regard to expense. The meeting proposed to adopt the report and to refer all matters arising out of it to the committee to be appointed.

Mr. Melvill Green said the Governors at the meeting in March determined that the expenses were to be borne as one or other of the two committees might determine, and both had determined that necessary and proper expenses should be allowed but the law charges were to be taxed if this meeting thought fit.

Mr. S. H. Bower (solicitor) stated that at the request of the Committee of Inquiry he had consented to allow his bill to be taxed.

Mr. F. Wigan stated that he was present on four or five occasions during the inquiry, and he could testify that it was conducted in a most admirable manner both by the gentlemen who represented the board and also by the medical staff themselves, and he did not think any Governor would grudge the expense of the inquiry.

Mr. Nelson asked if Mr. Rawlings had sent in his resignation to the board.

The Chairman stated that Mr. Rawlings had notified his intention to resign, and the matter would come before the next meeting of the board.

Mr. Nelson: It will be for the board to consider the advisability of accepting that resignation or not.

The Chairman: Yes.

Mr. Wigan: I cannot assent to that.

The Chairman added that in this matter as in others the board were in the hands of the Governors, by whose decision in general meeting they would undoubtedly feel bound; but speaking generally the board were in a position to accept or refuse the resignation.

Mr. Wigan said he understood that in adopting the Committee's report they accepted Mr. Rawlings's resignation.

The Chairman said the board a short time ago had a meeting with the Committee of Selection, and it was then arranged, after talking the matter over, that in order to bring about peace in the institution—a condition which had been absent from it for some time—it would be well, if the Governors thought proper, that the board should assent to the recommendation of the Committee of Inquiry, so far as it related, first of all, to the admission of two members of the medical staff to the board, and, secondly, to the abolition of the office of Secretary-Director. That, he took it, was the main part of the report. He thought that more blame had been attached to the board and perhaps to Mr. Rawlings than the Committee of Inquiry had cast upon them. Upon the whole the opinion of the Committee was that the condition of the hospital was good and that the management had been satisfactory. ("No, no.") In their report the Committee stated that they had paid two surprise visits to the hospital, and, generally speaking, the hospital was in good order and the patients to whom they spoke expressed themselves as satisfied with their food, treatment, and nursing. They also reported that they had inquired into the truth of the several allegations contained in the statement of the medical staff, and they found that some of the most important of them were true, that some had a foundation of fact but were exaggerated, and that others were unsupported by any sufficient evidence. Again, it was their opinion that the defects in management, to which attention was drawn in the statement of the medical staff, were deserving of notice and correction, but that they were never sufficient in magnitude and frequency to render the treatment in the hospital otherwise than good as a whole. No doubt the hospital, like other human institutions, had its failings. The board certainly did not profess to be immaculate; they did not claim that their management had been perfect in every respect. What they did claim was that the hospital was what it was in consequence largely of the action of the Board of Management. ("Oh" and laughter.) Some 25 or 30 years ago, when he was introduced to the institution by one of the founders, the charity was carried on in two little houses in Queen Square. Mr. Rawlings, of whom so much had been said, was then the Secretary of the institution. From that time to this the hospital had continued to advance, and the medical staff, at their festivals and elsewhere, had time after time expressed themselves as perfectly satisfied with the condition of the hospital. There was no hospital in the country which had advanced at a greater rate, or which perhaps had attained a greater name than theirs. No doubt that was largely due to the eminence of the medical staff, but the Board of Management claimed that some credit was due to them even for the appointment of that medical staff. He very much doubted if any institution could come out of such an inquiry as that which had been held with such a record as they had. ("Question.") He fully expected that the medical staff would have agreed with him on that point. ("No.") He admitted that there were defects of some sort, but such as they were they were not brought to the knowledge of the board. ("Oh, oh.") The medical staff ought, he thought, to have brought these matters before the Board of Management. (Voices: "We did.") All he could say was that he had attended the meetings of the board pretty regularly for the last 25 years, and these defects were not brought to his knowledge.

Mr. Victor Horsley, in the name of the medical staff, protested against the statement that they did not draw the attention of the board to the matters referred to. They had drawn the attention of the secretary-director to all these things, and yet the chairman had the audacity to say that they had not done so.

The Chairman said what he meant was that if these things had been brought before the board he did not see how he, as a constant attendant, did not know of them.

Mr. Victor Horsley: I must accept your explanation, but I do not think it is satisfactory.

Sir Sydney Waterlow appealed to the chairman to rule whether this discussion was in order on a motion for the appointment of a committee.

The Chairman replied that it was somewhat irregular, but he would like to read the 39th clause of the report. It was as follows: "The statement alleges that the Board of Management have refused to provide for the washing of sufficient bed linen, and that the attention of the secretary and general director has been directly drawn to the evils alleged in reference to the condition, care and treatment of the patients; but that no steps have been taken to remove them. These allegations have, in our judgment, not been proved."

Mr. Melvill Green suggested that the discussion should cease, remarking that they were not concerned with the past but with the future.

Sir James Crichton-Browne said the attitude taken up by the chairman, on behalf of the board, was unfair to Mr. Rawlings. The board had for years persistently shut its ears to all efforts of reform, and as the committee stated in their report, on them rested the responsibility for the attitude Mr. Rawlings assumed towards the medical staff. (Hear, hear.)

The motion, as amended, was then unanimously agreed to.

Mr. Melvill Green further moved, "That such Committee consist of nine persons, three of whom should be appointed by the Board of Management, two by the medical staff, and four by the committee appointed on March 23 last, and that four should form a quorum."

Sir Sydney Waterlow seconded the motion, which was carried unanimously.

Mr. Melvill Green announced that it was the intention of the Committee of Inquiry to appoint Mr. Timothy Holmes, Mr. J. Danvers Power, Mr. James Wigan, and himself, as members of the Committee. He understood the medical staff were prepared to nominate two members, but the board were not ready to nominate their members.

Dr. Bastian stated that the medical staff would be represented by Dr. Ormerod and Sir Felix Semon.

The Chairman said that unless the meeting strongly urged them to do so, the Board of Management would prefer not to appoint their representatives that day.

Mr. WIGAN considered it would be more satisfactory if the board announced at once the names of the gentlemen whom they intend to appoint.

The CHAIRMAN stated that the board believed that a gentleman who had had great experience in hospital management might be induced to join the Committee, but he had not yet given his consent, and therefore he would name Mr. Colin Campbell, Mr. G. W. E. Russell, and himself.

A vote of thanks to the chairman terminated the proceedings.

